# The Correlation of Serum Metal Ions with Functional Outcome Scores at Three-to-Six Years following Large Head Metal-on-Metal Hip Arthroplasty

**DOI:** 10.1155/2013/173923

**Published:** 2013-03-27

**Authors:** Sheethal Prasad Patange Subbarao, Ibrahim A. Malek, Khitish Mohanty, Phillip Thomas, Alun John

**Affiliations:** Department of Orthopaedics, University Hospital of Wales, 84 Colchester Avenue Penylan, Cardiff, UK

## Abstract

Based on success of hip resurfacing, large head Metal on Metal (MoM) hip arthroplasty has gained significant popularity in recent years. There are growing concerns about metal ions related soft tissue abnormalities. The aim of this study was to define a correlation of metal ions with various functional outcome scores following large head MoM hip arthroplasty. Consecutive cohort of 70 patients (76 hips) with large head MoM hip arthroplasty using SL-Plus femoral stem and Cormet acetabular component were prospectively followed up. An independent observer assessed the patients which included serology for metal ion levels and collection of Oxford Hip, Harris hip, WOMAC, SF-36 & modified UCLA scores. Median serum cobalt and chromium levels were 3.10 *μ*g/L (0.35–62.92) and 4.21 *μ*g/L (0.73–69.27) with total of median 7.30 *μ*g/L (2.38–132.19). The median Oxford, Harris, WOMAC, SF-36 and modified UCLA scores were 36 (6–48), 87 (21–100), 36 (24–110), 104 (10–125), and 3 (1–9), respectively. Seventeen patients had elevated serum cobalt and chromium levels ≥7 *μ*g/L. There was no significant correlation between serum metal ion levels with any of these outcome scores. We recommend extreme caution during follow up of these patients with large head MoM arthroplasty.

## 1. Introduction

Following the success of hip resurfacing procedures, large head metal-on-metal hip (MoM) arthroplasty has become popular due to perceived advantages of low wear rate of metal-on-metal bearings and reduced dislocation rate of utilising large heads (≥36 mm). Midterm clinical results of such device have been encouraging [1–6]. Systemic effects of metal ions and wear debris of metal-on-metal bearings leading to necrotic and inflammatory local soft tissue reactions are cause of concern [7]. Medicine and Healthcare products Regulatory Agency (MHRA) issued alert on most MoM bearings suggesting continued surveillance of such device with regular clinical outcome, metal ion level measurement and cross-sectional imaging if required [8]. 

There is ongoing debate about safe level of metal ions, relation to soft tissue abnormality, and its use in screening for MoM-bearing device followup. De Smet et al. recommended that metal ion measurement should be used as diagnostic tool to identify problems with MoM hip resurfacing based on their work of revision surgery for metal-on-metal bearing device [9]. It is now understood wear characteristics of large head MoM hip implants are significantly different and higher than those of resurfacing implants [10–12]. 

There are outcome scores that are routinely used in all prospective and retrospective studies. Harris hip score is most widely used in literature [13]. Oxford hip score, Western Ontario and McMaster Universities Osteoarthritis Index (WOMAC) and Short Form SF-36 health survey, and Modified University of California at Los Angeles (UCLA) score are few of the many scoring systems used for objective assessments following hip arthroplasty [14–17]. However there is very little evidence in literature demonstrating statistical correlation between the outcome scores and the individual quantitatively measured values like serum metal ions. Thus there is an argument that whether there is a ceiling effect that is reached with such scoring systems, to determine subtle differences when comparing different bearing surfaces. 

The purpose of this study was to ascertain the statistical correlation between commonly used different functional outcome scores for total hip arthroplasty and the serum chromium and cobalt ions levels, as a predictor of adverse reaction to metal debris (ARMD) leading to failure of MoM hip replacements.

## 2. Methods

Between June 2004 and August 2007, seventy patients underwent large head MoM total hip replacement using SL-Plus stem (Plus Orthopaedics, Smith & Nephew, UK) and Cormet (Corin Group Plc, Cirencester, UK) acetabular components. All patients were operated by orthopaedic consultants in the University Hospital of Wales Cardiff UK with special interest in lower limb arthroplasty. 

Patients were invited to attend the follow-up clinic run by an independent nurse practitioner. Patients were requested to complete a detailed questionnaire and provide blood sample for metal ion analysis. A combination of clinician-based and patient-based as well as disease-specific and generic health status measure clinical outcome scores was utilised. The questionnaire consisted of Oxford hip score, Harris hip score, WOMAC score, SF-36, and modified UCLA score.

### 2.1. Metal Ion Serology

An informed verbal consent was obtained before obtaining the blood sample. 5 mL of venous blood was obtained from the ante cubital fossa using 21-gauge needle connected to Vacutainer (Vacuette, Greiner Bio-One GmbH, Austria) in tubes containing trace element of sodium ethylene diaminetetra acetic acid (EDTA). Samples were sent immediately to the laboratory for analysis. The serum metal ion analysis was carried out at Biochemistry Department, Cardiff University Hospital, Cardiff, which is a participating laboratory in the trace elements external quality assessment scheme (TEQAS) as per MHRA guidance UK. Serum levels of cobalt and chromium were measured with the use of an inductively coupled plasma mass spectrometry technique (ELAN DRC II; PerkinElmer Life and Analytical Sciences, Waltham, MA, USA). The serum Cobalt and Chromium levels ≥7 *μ*g/L for either cobalt or chromium were considered elevated as suggested by Medicine and Healthcare products Regulatory Agency (MHRA) UK. 

Statistical analysis was carried out using SPSS version 17.0 for Windows (SPSS Inc., Chicago, IL, USA). The Spearman rank's two tailed test at the significance level of 0.01 was utilised to derive correlation between outcome scores and serum cobalt and chromium levels.

## 3. Results

Seventy-six large head MoM hip replacements were performed in seventy patients between 2004 and 2008. There were 37 males and 33 females with average age of 58 years (range: 18–77 years) at the time of surgery. Mean followup was 51 months (Range: 36–74 months). All patients presented for followup and there were no deaths reported.

The median oxford hip score was 36 (range: 6–48) and Harris hip score was 87 (Range: 21–100). The median WOMAC score was 36 (range: 24–110), SF-36 score was 104 (range: 10–125), and modified UCLA score was 3 (range: 1–9). Mean serum cobalt level was 3.10 *μ*g/L (range: 0.35–62.92) and serum chromium level was 4.21 *μ*g/L (range: 0.73–69.27). 

**Figure 1:**
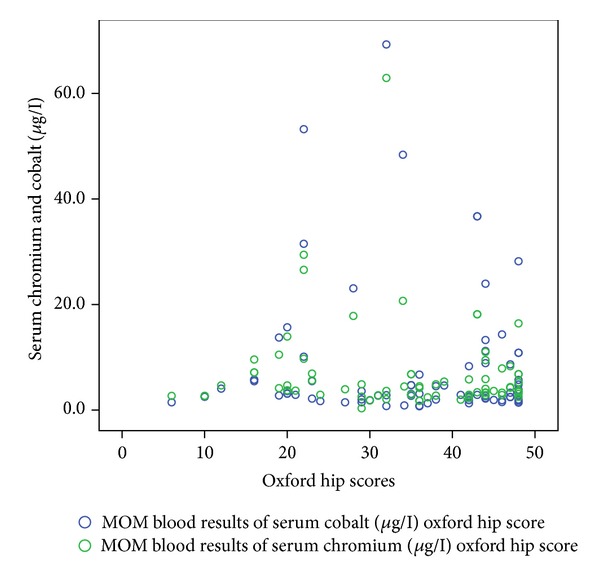
The correlation of Oxford hip score versus serum cobalt and chromium levels.

**Figure 2:**
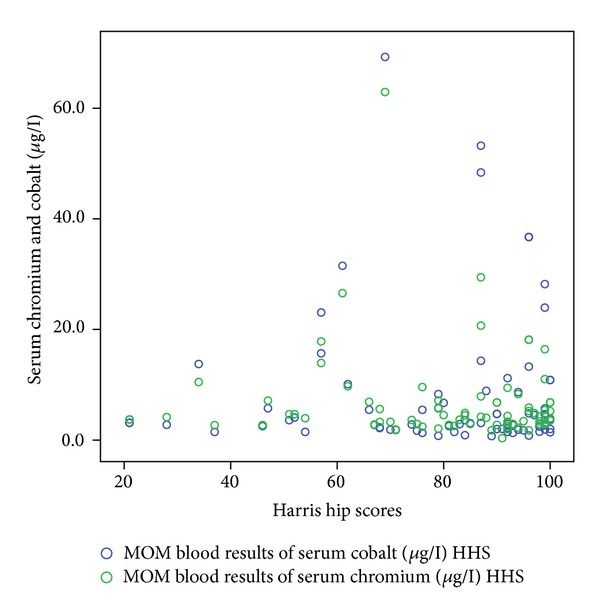
The correlation of harris hip score versus serum cobalt and chromium levels.

**Figure 3:**
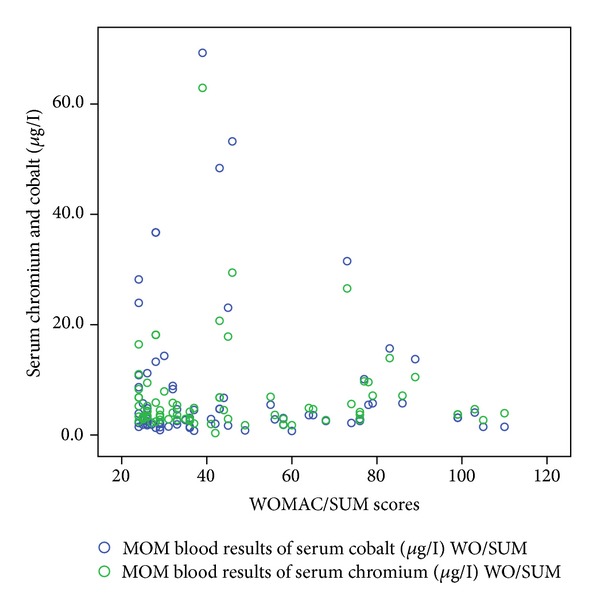
The correlation of WOMAC score versus serum cobalt and chromium levels.

**Figure 4:**
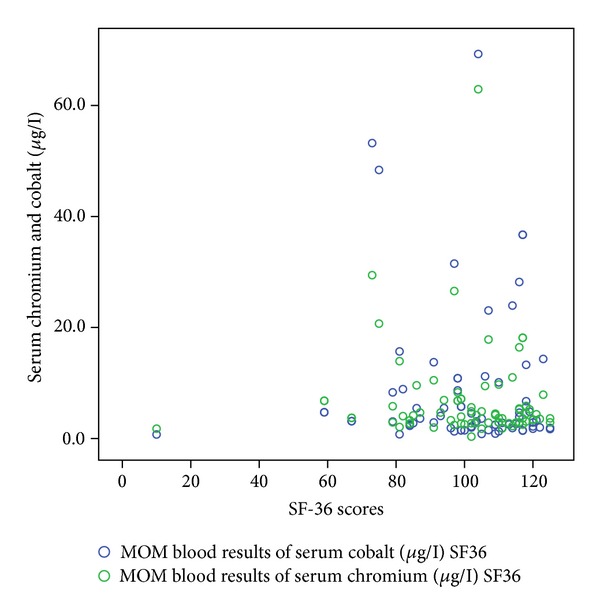
The correlation of SF-36 score versus serum cobalt and chromium levels. Serum cobalt and chromium level.

**Figure 5:**
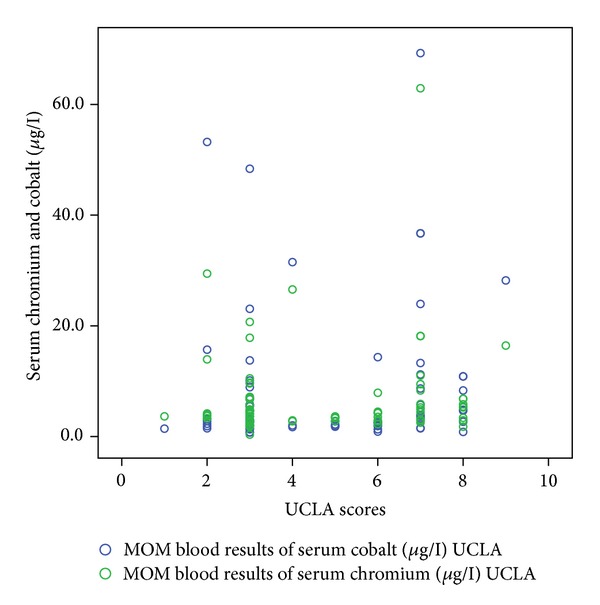
The correlation of modified UCLA score versus serum cobalt and chromium levels.

Table 1 shows the correlation coefficient and *P* values of these functional outcome scores with serum cobalt and chromium levels. Figures 1, 2, 3, 4, and 5 demonstrate scatter-plot diagrams of above functional outcome scores versus serum cobalt and chromium levels.

Fourteen patients were further investigated with cross sectional imaging in the form of metal artefact reduction sequence (MARS) MRI scans June 2011, and six scans have been positive for ARMD.

## 4. Discussion

There are various outcomes score used following a total hip arthroplasty [13–19]. These outcome scores use modalities such as pain, quality of life, activities, and range of motion. There is an assumption that, with all outcome scores, postoperatively there is a general improvement. Failure of an improved score is assumed to have a poor outcome. The scoring systems used provide an idea of activity, pain level, functional characteristics, disease-specific improvements pre- and postoperatively. They are also affected by the existing comorbidities. There is now growing trend to follow up these patients in a designated arthroplasty clinic often led by extended scope nurse practitioner nurse or physiotherapist, once patients have made an uneventful postoperative recovery.

Reports of increasing incidence of ARMD in patients with MoM-bearing hip device is a huge concern [20–24]. After a peak in the middle of last decade, there has been a fall in the trend of using MoM devices following the adverse reaction as suggested in the National Joint Registry 7th annual report [25]. The MHRA UK issued a medical device alert because of increasing concerns regarding these bearings in April 2010 [8]. A cutoff of 7 ppb was recommended above which there is a need for clear observation and revision surgery if indicated. Most recently, a document from the British Hip Society has stated that Blood Cobalt and Chromium ions are often, but not always elevated [26]. It is paramount that these patients are regularly followed up with metal ions serology. We are still in primacy of understanding of pathophysiology and clinical presentation of ARMD, and a lot more will be revealed in future about the true extent of damage. It was initially thought that patients with such locally invasive pathology will be clinically symptomatic. A recent study has raised a huge concern of presence of ARMD in asymptomatic patients [27, 28]. 

The establishment of a surveillance programme for these patients is not an easy task especially for small-to-medium sized district general hospitals. Most of these hospitals do not have the facility to conduct an expensive metal ions serology, and they have to send these samples to a larger laboratory participating in the Trace Elements External Quality Assessment Scheme (TEQAS) as per MHRA requirement. The investigating clinician can misleadingly be reassured with good-to-excellent postoperative outcome scores and routine radiographs which are often unremarkable even in the presence of significant ARMD. Only three out of seventeen patients in our cohort had lower outcome scores and remaining were well-functioning hips with good functional outcome scores. The lack of significant correlation between various functional outcome scores and serum metal ions can be explained by following reasons. First, a significant number of patients with ARMD are asymptomatic or the functional outcome measures are not sensitive enough to detect small changes in general health due to ceiling effect which is a well known limitation like Harris hip score. Our results suggest that even more detailed functional outcome measures like WOMAC and SF 36 scores also failed to show significant correlation with serum metal ions.

We recognise the limitations of our study. There were no preoperative functional outcome scores available for comparison with postoperative scores. There were six patients with bilateral MoM hip replacements. We did not separately study the correlation characteristics between the metal ions levels and scoring pattern in bilateral MoM hip replacements. The blood level of cobalt and chromium ions may vary in case of bilateral hip replacements. There is no separate guidance from MHRA about bilateral prosthesis. It is also assumed that serum metal ions do not have a diurnal variation. There was no standardisation of physical activity before the samples were taken in the clinics, that is, resting or fasting. So far, we are not aware of immediate effect of activity on serum metal ion levels. It is also possible that these results from a specific implant may not be applicable to other manufacture implant brands as sometimes they have different wear characteristics [29]. 

It is now also understood that ARMD is a progressive disorder, and revision procedures have poor outcome especially if deferred [30, 31]. Our results identify that patients with MoM hip replacement frequently have an elevated serum metal ions despite well-functioning hips which might represent silent ARMD. We recommend an extremely vigilant strategy with these patients, which requires the education of allied medical staff involved in follow-up clinics, detailed clinical examination, metal ion serology, and readily accessible radiology service for cross-sectional imaging. The regular use of functional outcome scores remains an extremely important tool to assess and report outcomes following total hip arthroplasty but the clinicians should be extremely careful in relying solely on those for implementation of further management of patients with MoM hip replacements.

## Figures and Tables

**Table 1:** Correlation coefficient of functional outcome scores versus the serum cobalt and chromium metal ion levels.

	Oxford hip score	Harris hip score	WOMAC	SF-36	UCLA
Serum cobalt	−0.038	0.001	0.0001	−0.093	0.173
	(*P* = 0.743)	(*P* = 0.990)	(*P* = 0.999)	(*P* = 0.424)	(*P* = 0.135)
Serum chromium	−0.044	−0.010	0.029	−0.057	0.117
	(*P* = 0.704)	(*P* = 0.930)	(*P* = 0.806)	(*P* = 0.623)	(*P* = 0.312)
